# In vitro and in silico evaluation of flavonoids from *Erythrina crista-galli* with cytotoxic potential against MCF-7 breast cancer cell

**DOI:** 10.1038/s41598-025-32400-4

**Published:** 2025-12-18

**Authors:** Tati Herlina, Melati Sukma, Abd. Wahid Rizaldi Akili, Ari Hardianto, Jalifah Latip, Euis Julaeha, Tri Mayanti

**Affiliations:** 1 Department of Chemistry, Faculty of Mathematics and Natural Science, Universitas Padjadjaran, Jatinangor, Sumedang, West Java 45363 Indonesia; 2 Department of Chemical Sciences, Faculty of Science and Technology, Universiti Kebangsaan Malaysia (UKM), 46300 Bangi, Selangor Malaysia

**Keywords:** Erythrina crista-galli, Flavonoids, Cytotoxic, In vitro, In silico, Biochemistry, Cancer, Chemistry, Computational biology and bioinformatics, Drug discovery

## Abstract

Naturally occurring flavonoids have garnered significant interest as potential anticancer agents owing to their structural diversity and ability to modulate multiple molecular targets. This study aimed to investigate the cytotoxic potential of flavonoids isolated from *Erythrina crista-galli* in MCF-7 breast cancer cells using in vitro and in silico analyses. Three flavonoids were isolated from the twigs of *E. crista-galli* using multiple column chromatography. Cytotoxic activity was evaluated against the MCF-7 breast cancer cell line using the MTT assay, whereas the in silico study was evaluated using molecular docking and molecular dynamics against the epidermal growth factor receptor (EGFR). Three flavonoids, naringenin (1), daidzein (2), and isoliquiritigenin (3), were isolated from the ethanol extract of *E. crista-galli* twigs. Their structures were confirmed using multiple spectroscopic methods. In vitro cytotoxicity testing against MCF-7 breast cancer cells showed that naringenin (1) and isoliquiritigenin (3) exhibited moderate activity, with IC_50_ values of 105.60 and 84.21 µg/mL, respectively, whereas daidzein (2) displayed weak activity, with IC_50_ of 239.77 µg/mL. Network pharmacology analysis identified multiple targets of isoliquiritigenin related to breast cancer, with epidermal growth factor receptor (EGFR) as one of the key hub. Molecular docking indicated a stronger binding affinity of isoliquiritigenin compared to ATP, while molecular dynamics simulations confirmed the stability of the EGFR–isoliquiritigenin complex with binding free energy estimated at − 30.002 ± 3.879 kcal/mol. Collectively, these results highlight isoliquiritigenin as a promising EGFR-targeted cytotoxic compound and *E. crista-galli* as a valuable source of bioactive flavonoids for anticancer drug development, while emphasizing the need for further in vivo validation and toxicity assessment.

**Supplementary Information:**

The online version contains supplementary material available at 10.1038/s41598-025-32400-4.

## Introduction

Cancer, a disease characterized by uncontrolled growth of transformed cells that evolve under natural selection^1^, is considered a major burden of disease worldwide and the second most common cause of death globally^2^. The number of cancer case is expected to increase over the next two decades. In 2020 there were approximately 18.1 million new cases of cancer were reported worldwide, with breast, lung, bowel, and prostate cancers collectively account for 43% of all the new diagnoses^3^. In terms of breast cancer, 2.3 million new cases and 670,000 deaths were recorded in 2022. These numbers are projected to increase by 38% and 68% by 2050, particularly impacting low Human Development Index (HDI) countries, highlighting the need for early diagnosis and better access to treatment^4^.

To date, various of cancer treatments are available, including surgery, chemotherapy, radiotherapy, and immunotherapy. The success rate and whether the therapy is used alone or in combination with others depend on the type and stage of the cancer. These treatments also come with their own drawbacks. Surgery and radiotherapy have a local effect, whereas chemotherapy has systemic effect^5^. The use of chemotherapeutic agents has been reported to cause side effects. For example, doxorubicin is known to exhibit cardiotoxic effects in a large number of patients^6^. Another side effects, such as fatigue, loss of appetite, and diarrhea were reported on patients during treatment with chemotherapeutic agents^7^. Additionally, over time, cancer cells can develop resistance to chemotherapeutic agents through multiple mechanism such as altering drug targets, enhancing drug efflux, improving DNA repair and reducing apoptosis, leading to reduced treatment effectiveness^8^. To overcome these challenges, discovery and development of new drugs are needed, particularly those derived from natural products, which are more accessible and generally less toxic^9^.

Throughout history, plants have provided humans with bioactive compounds for medicinal purposes^10^. Nowadays, many of these phytochemicals have been studied as potential anticancer drugs^11^. Flavonoids are among groups of phytochemicals that are generally considered safe, and based on numerous studies, they exhibit anticancer potential through multiple mechanisms, including, inhibit cellular proliferation, induce apoptotic and autophagic cell death, induce cell cycle arrest and modulate the antioxidant enzymes^12^. These properties highlight their role as potential anticancer drug. *Erythrina crista-galli* is one of the plant species belonging to the genus *Erythrina*, which is recognized as a natural source of structurally diverse flavonoids. However, studies focusing on cytotoxic potential, particularly on this species remain scarce^13^. Therefore, this study aimed to isolate flavonoids from twigs of *E. crista-galli* and to evaluate their cytotoxic potential using in vitro and in silico studies.

## Materials and methods

### Plant materials

Twigs of *E. crista-galli* were collected from the Sersan Badjuri St., Bandung, West Java, Indonesia. These plant materials were determined at the Laboratory of Agricultural Production Technology & Services, Agricultural Cultivation Department, Faculty of Agriculture, University Padjadjaran, under voucher specimen number 1020 and was identified by Joko Kusmoro.

### Extraction and fractionation

Powdered twigs of *E. crista-galli* (2 kg) were subjected to ultrasonic-assisted extraction using ethanol as the solvent. Extraction was performed for 29.5 min at 37 °C with a sample-to-solvent ratio of 1:19 (w/v). The resulting extract was filtered and concentrated under reduced pressure using a rotary evaporator at approximately 35 °C, yielding 170 g crude ethanol extract. The crude extract was partitioned with n-hexane, ethyl acetate, and butanol. Each fraction was then concentrated under the same conditions to obtain 12 g n-hexane extract, 30 g ethyl acetate extract, and 21 g butanol soluble fraction. 

### Flavonoids isolation

A total of 30 g of the ethyl acetate soluble fraction was subjected to vacuum liquid chromatography (VLC) using n-hexane: ethyl acetate: ethanol as the eluent with a 10% gradient, yielding eight main fractions (A–H). Fraction D (1.1 g) was further separated by open column chromatography with n-hexane: ethyl acetate (5% gradient), affording ten subfractions (D1–D10). Subfraction D5 (80 mg) was fractionated by open-column chromatography using chloroform: ethyl acetate (5% gradient) to obtain subfraction D5d (50 mg). This fraction was further separated by normal-phase chromatography using chloroform: ethyl acetate (8:2) to afford fraction D5d3 (22 mg). Subsequent purification via normal-phase chromatography using n-hexane: chloroform: ethyl acetate (2:7:1) yielded D5d3a (12 mg). Final purification was carried out by reversed-phase column chromatography using methanol: water (7:3), affording compound 1 (3.1 mg).

Subfraction D7 (210 mg) was separated via normal-phase chromatography using methylene chloride: ethyl acetate (8:2), affording fraction D7d (107 mg). This fraction was further separated by normal-phase chromatography with chloroform: ethyl acetate (8:2), yielding fraction D7d2 (38.2 mg). Purification of this fraction by reversed-phase column chromatography using methanol: water (6:4, gradient 5%) yielded two pure compounds, compound 2 (3.2 mg) and compound 3 (8.2 mg). 

### Cytotoxic assay

MCF-7 (ATCC, HTB-22) cancer cells were sourced from central laboratory of Universitas Padjadjaran. MCF-7 cells were seeded in 96-well microplates at a density of 3 × 10^4^ cells per well and incubated for 24 h at 37 °C in a humidified atmosphere containing 5% CO_2_. The test samples were dissolved in DMSO to prepare 2000 µg/mL stock solution. The sample were serially diluted in culture medium to obtain range of concentration for testing (7.81–1000 µg/mL). After 24 h incubation, 100 µL of each test sample was added to each well. The plates were then incubated for another 24 h under the same condition. Following the treatment, the medium was discarded and 100 µL of MTT cell viability reagent was added to each well. The plates were incubated for 1 h, and the absorbance was measured at 570 nm using multimode plate reader. The IC_50_ was calculated by the percentage of viable cells relative to untreated control and is defined as the concentration required to inhibit cell growth by 50%. To ensure reproducibility, the assay was performed in duplicate with cisplatin as positive control. 

### Network pharmacology

The 2D structure of the active compound was uploaded to SwissTargetPrediction (http://www.swisstargetprediction.ch/), and the species was set to *Homo sapiens* to obtain predicted molecular target. Breast cancer-related genes were retrieved from the GEPIA 2 (https://gepia2.cancer-pku.cn/) and Gene Expression Omnibus (https://www.ncbi.nlm.nih.gov/geo/), and the overlapping genes between the disease-associated genes and compound-predicted targets were identified. The shared protein targets were imported into STRING database (https://string-db.org/) to generate a protein-protein interaction (PPI) network using a high confidence score threshold (> 0.7). Pathway enrichment analysis of the intersecting targets was performed using Kyoto Encyclopedia of Genes and Genomes (KEGG) database (https://www.genome.jp/kegg/). To further identify biological pathways associated with the compound’s potential bioactivity, the ShinyGO 0.85 database was used for complementary enrichment analysis (https://bioinformatics.sdstate.edu/go/). 

### Molecular docking

The protonation state of the isolated compound at physiological pH (7.4) was predicted using Chemaxon MarvinSketch. A three-dimensional (3D) structure of compound 1 was generated and energy-minimized using the Merck Molecular Force Field (MMFF94). For docking preparation, the structure was refined by adding hydrogen atoms, assigning Gasteiger charges, and defining rotatable bonds.

The 3D X-ray crystal structure of the epidermal growth factor receptor (EGFR) kinase domain (PDB ID: 3W32) was obtained from the Protein Data Bank (https://www.rcsb.org, accessed on 16 April 2025). The co-crystallized ligand was separated from the receptor and explicit water molecules were removed to obtain the apo form of the protein. Hydrogen atoms and charges are subsequently added to the receptor structure. Finally, both the prepared protein and the co-crystallized ligand were subjected to a redocking procedure using AutoDock Vina version 1.2.0. The grid box dimension used for the redocking procedure was 40 × 40 × 40 with 0.375 Å spacing, and grid center coordinate of 16.551 (x), 33.325 (y), 12.457 (z). The redocking procedure yielded RMSD value of 1.6323 Å, suggesting validity of the docking parameters^14^.

### Molecular dynamics simulation

Molecular dynamics simulation was conducted as described in our previous study^15,16^. Before the simulation, histidine residues in the protein structure were refined using the pdb4amber program, where their protonation states were adjusted based on local chemical environments, and the system was parameterized using the ff19SB force field. Ligand partial charges were calculated using the Austin Model 1-Bond Charge (AM1-BC) method implemented in the antechamber tool of AmberTools21, and the ligands were further parameterized using the Generalized Amber Force Field 2 (GAFF2). The ligand–protein complex was then assembled using a tleap from AmberTools21. Solvation was performed using the TIP3P explicit water model within a rectangular box extending 10 Å from the solute. Counter ions (Na^+^ and Cl^−^) were added to neutralize the system and reach a physiological salt concentration of 0.15 M.

Molecular dynamics simulations were performed using the particle mesh Ewald Molecular Dynamics (PMEMD) module of AMBER20 with GPU acceleration. Energy minimization of the ligand–protein complex was carried out in two stages: an initial restrained minimization comprising 1000 steps of steepest descent, followed by 2000 steps of conjugate gradient minimization under a harmonic force constant of 5 kcal·mol⁻¹·Å⁻², and a subsequent unrestrained conjugate gradient minimization of 5000 steps to resolve any steric clashes. The system was then gradually heated from 0 to 300 K in three stages (0–100, 100–200, and 200–300 K) over 20 ps, for a total of 60 ps. Equilibration was performed for 1000 ps to stabilize the density and pressure, while progressively releasing positional restraints. Finally, a production run of 100 ns was conducted with an integration time step of 2 fs. Binding energy calculation was performed using the MMGBSA.py module in AmberTools20^17^.

### In silico toxicity and pharmacokinetics prediction

The toxicity and pharmacokinetics of the compounds were predicted using the pkCSM web server (https://biosig.lab.uq.edu.au/pkcsm/; accessed May 12, 2025). 

## Result and discussion

### Structure elucidation of compound 1–3

Compound 1 was obtained as a yellow, amorphous powder. The HR-TOFMS spectrum revealed that compound 1 had *m/z* of 273.0753 [M + H]^+^ (calculated as 273.0763 for C_15_H_13_O_5_^+^). ^1^H-NMR (700 MHz, CDCl_3_) δ_H_ (ppm): 5.40 (1H; dd; *J* = 3.0; 13.0 Hz, H-2), 2.80 (1H, dd, *J* = 2.9; 16.8 Hz, H-3a), 3.04 (1H, dd; *J* = 13.2; 16.8 Hz, H-3b), 6.42 (1H, d, *J* = 2.4 Hz, H-6), 6.43 (1H; d; *J* = 2.4 Hz, H-8), 7.35 (21 H; dd; *J* = 8.6; 2.4 Hz, H-2` and H6`), 6.87 (2 H; dd; *J* = 8.6; 2.4 Hz, H-3` and H-5`), 13.44 (1H; s). ^13^C-NMR (175 MHz, CDCl_3_) δ_C_ (ppm): 79.9 (C-1), 44.3 (C-3), 190.2 (C-4), 162.8 (C-5), 103.0 (C-6), 162.4 (C-7), 110.0 (C-8), 163.0 (C-9), 114.7 (C-10), 130.0 (C-1`), 128.5 (C-2`), 115.9 (C-3`), 156.3 (C-4`), 115.9 (C-5`), and 127.9 (C-6`). The NMR spectrum of compound 1 is consistent with that of naringenin reported in the literature^18^.

Compound 2 was obtained as dark-yellow oil. The HR-TOF-MS spectrum revealed that compound 2 had *m/z* of 255.0647 [M + H]^+^ (calculated 255.0657 for C_15_H_11_O_4_^+^). ^1^H-NMR (700 MHz, CDCl_3_) δ_H_ (ppm): 8.53 (1H, s, H-2), 7.90 (1H, dd, *J* = 8.3; 2.4 Hz, H-5), 7.04 (1H, dd, *J* = 8.3; 2.4 Hz, H-6), 6.83 (1H, s, H-8), 7.53 (2 H; d; *J* = 8.6 Hz, H-2` and H-6`), 6.83 (2 H; d; *J* = 8.6 Hz, H-3` and H-5`). ^13^C-NMR (175 MHz, CDCl_3_) δ_C_ (ppm): 139.5 (C-2), 127.6 (C-3), 176.1 (C-4), 111.3 (C-4a), 127.9 (C-5), 115.9 (C-6), 166.8 (C-7), 103.5 (C-8), 159.2 (C-8a), 127.8 (C-1`), 130.5 (C-2`), 115.6 (C-3`), 168.9 (C-4`), 115.6 (C-5`), 130.5 (C-6`). The NMR spectrum of compound 2 is consistent with that of daidzein reported in the literature^19^.

**Fig. 1:**
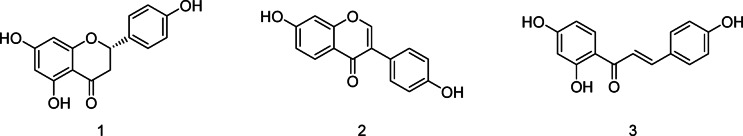
Chemical structure of compound 1–3.

Compound 3 was obtained as a light-yellow amorphous powder. The HR-TOFMS spectrum revealed that compound 3 had *m/z* of 257.0820 [M + H]^+^ (calculated as 257.0814 for C_15_H_13_O_4_^+^). ^1^H-NMR (700 MHz, CDCl_3_) δ_H_ (ppm): 7.53 (1H, d, *J* = 15.2 Hz, H-1), 7,74 (1H, d, *J* = 15.2 Hz, H-2), 6,23 (1H, d, *J* = 2.3 Hz, H-3`), 6,36 (1H, dd, *J* = 8.6; 2.3 Hz, H-5`), 7,99 (1H, d, 8.6), 7.53 (2 H, d, *J* = 8.6 Hz, H-2`` and H-6``), 6.86 (2 H, d, *J* = 8.6 Hz, H-3``and H-5``). ^13^C-NMR (175 MHz, CDCl_3_) δ_C_ (ppm): 118.9 (C-1), 146.9 (C-2), 195.0 (C-3), 114.2 (C-1`), 166.4 (C-2`), 103.8 (C-3`), 168.1 (C-4`), 109.3 (C-5`), 133.3 (C-6`), 127.8 (C-1``), 131.9 (C-2`` and C-6``), 116.7 (C-3`` and C-5``), 161.6 (C-4``). The NMR spectrum of compound 3 is consistent with that of isoliquiritigenin reported in the literature^20^. The structures of compound 1–3 are given in Fig. 1.

### In vitro cytotoxic assay

**Fig. 2:**
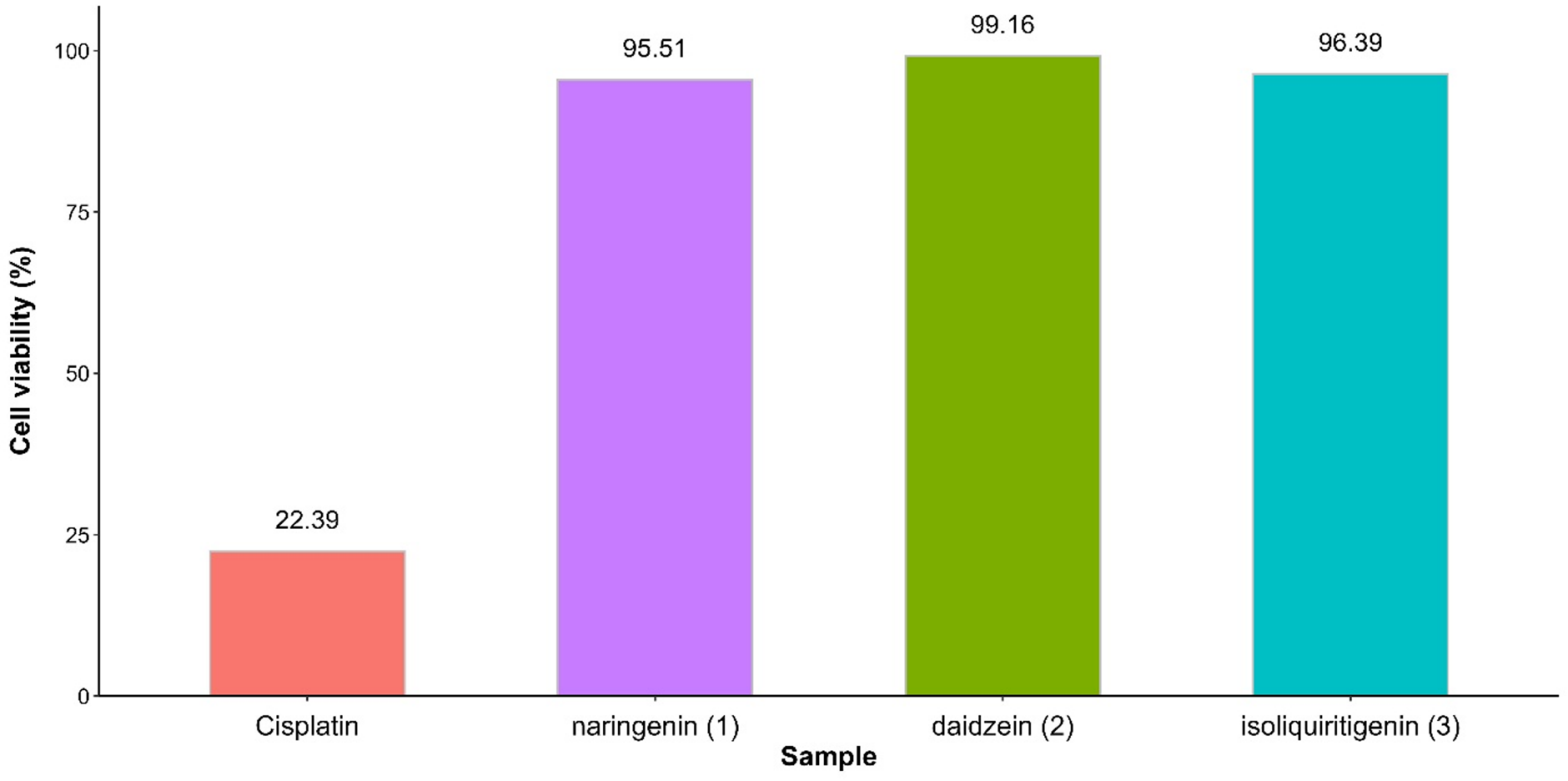
Cytotoxic comparison of cis-platin (12.96 µg/mL) with naringenin (1), daidzein (2), and isoliquiritigenin (3) at 15.63 µg/mL.

The cytotoxic activity of the isolated flavonoids against MCF-7 breast cancer cells was evaluated using an MTT assay. Cytotoxic comparison showed that cisplatin as the positive control exhibited the highest cytotoxic effect with 22.39% cell viability at 12.96 µg/mL, highlighting its established efficacy as a chemotherapeutic agent. In contrast, naringenin (1), daidzein (2), and isoliquiritigenin (3) showed relatively lower cytotoxic effect at the concentration of 15.63 µg/mL (Fig. 2). Furthermore, the cytotoxic effect of the isolated compounds showed dose-dependent manner (Figure S1–S3), with isoliquiritigenin (3) exhibiting the strongest cytotoxic effect having an IC_50_ value of 84.31 µg/mL, followed by naringenin (1) with an IC_50_ value of 105.60 µg/mL. Daidzein (2) showed the weakest activity, with an IC_50_ of 239.77 µg/mL. In line with the present results, isoliquiritigenin (3) has been reported to exert cytotoxic activity against several cancer cell lines, including liver^21^, breast, prostate^22^, and cervical cancer cells^23^.

### Network pharmacology

**Fig. 3:**
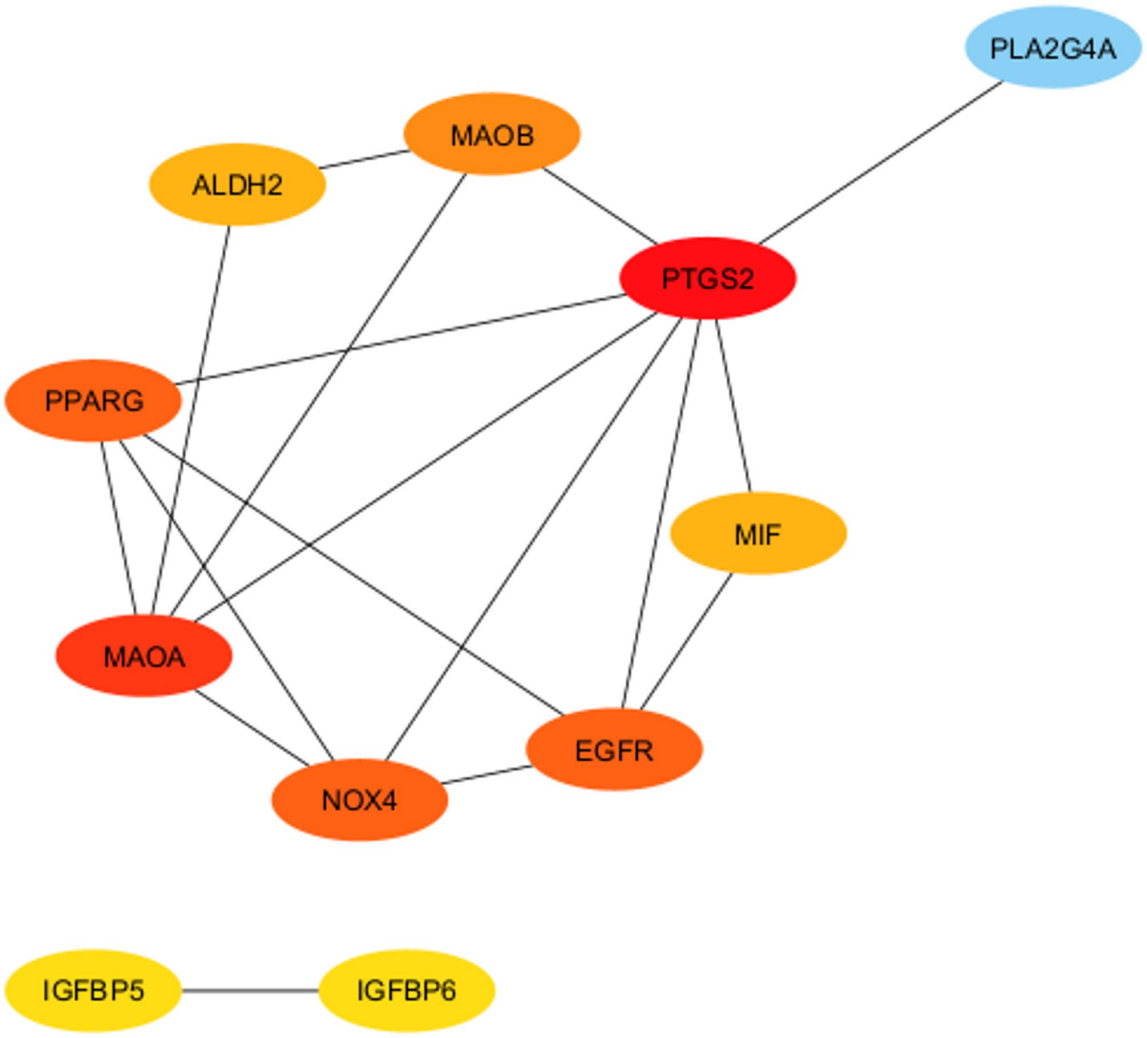
Target genes of isoliquiritigenin on breast cancer analyzed by Cytoscape.

The in vitro cytotoxicity assay revealed that isoliquiritigenin exhibited the highest cytotoxic activity against the MCF-7 breast cancer cell line. Given the polypharmacological nature of natural products, which allow them to interact with multiple targets^24^, network pharmacology was conducted as a complementary study to predict the possible interactions of isoliquiritigenin with breast cancer-associated genes. A total of 1,888 gene targets associated with breast cancer were retrieved from GEPIA 2 and Gene Expression Omnibus, and 84 potential targets for isoliquiritigenin were obtained from swiss target prediction. Intersection of these datasets yielded 16 common targets, which were considered the core targets mediating the potential anticancer effects of isoliquiritigenin against breast cancer. Protein-Protein Interaction (PPI) revealed 11 hub genes, including PTGS2, MAOA, EGFR, NOX4, PPARG, MAOB, ALDH2, MIF, PLA2G4A, IGFBP5, and IGFBP6 as central hub genes with the highest degree value (Fig. 3). This suggests that isoliquiritigenin may exert its cytotoxic activity through multiple pathways, including inflammation (PTGS2, PLA2G4A, MIF), proliferation and cell survival (EGFR, IGFBP5, IGFBP6), oxidative stress (MAOA, NOX4, MAOB, ALDH4), and metabolic regulation (PPARG).

Furthermore, the gene ontology (GO) enrichment analysis showed a number of significantly enriched biological processes (BP), molecular function (MF), and cellular components (CC) among the differentially expressed genes. In terms of BP, the therapeutic targets mainly involved in prostaglandin biosynthesis and metabolism, fatty acid transport, cell proliferation, and response to xenobiotics stimuli (Fig. S4). The GO cellular component analysis revealed that the therapeutic targets were predominantly located in insulin-like growth factor related protein complexes, organelle membrane, mitochondrial compartments, and the endoplasmic reticulum (Fig. S5). For molecular function, the targets were mostly enriched in the activity of oxidoreductase, monoamine, growth binding factors, and receptor-related functions (Fig. S6). Kyoto Encyclopedia of Genes and Genomes (KEGG) pathway analysis revealed various significantly enriched pathways among the differentially expressed genes, which are including amino acid metabolism pathways, inflammation and lipid mediator biosynthesis, and cancer related pathways.

From the KEGG pathway analysis^25^, several genes, including EGFR, were involved in the cancer related pathways of isoliquiritigenin (Fig. S7). EGFR is recognized as a key therapeutic target in various cancers, including breast cancer, owing to its critical role in tumor growth and progression^26^. Overexpression of EGFR in breast cancer has been correlated with a larger tumor size, poor differentiation, and unfavorable clinical outcomes^27^. Considering the important role of EGFR, and the result of network pharmacology, EGFR was selected as target protein for molecular docking and molecular dynamics simulation.

### Molecular docking and molecular dynamics simulation

Molecular docking was performed to evaluate the interaction between isoliquiritigenin and EGFR. The docking results showed that isoliquiritigenin bound to EGFR with a docking score of −8.41 kcal/mol. This affinity was weaker than that of the co-crystallized inhibitor (−12.08 kcal/mol), which is expected because the reference ligand was specifically designed to form multiple optimized interaction within the EGFR ATP-binding pocket. In contrast, isoliquiritigenin is small, naturally occurring flavonoids with simpler scaffold and fewer functional groups compared to the reference ligand available for high-affinity contacts. Despite this inherent structural limitation, its docking score remained stronger than that of ATP (−7.51 kcal/mol), suggesting a higher binding affinity of isoliquiritigenin than the substrate of EGFR.

**Fig. 4:**
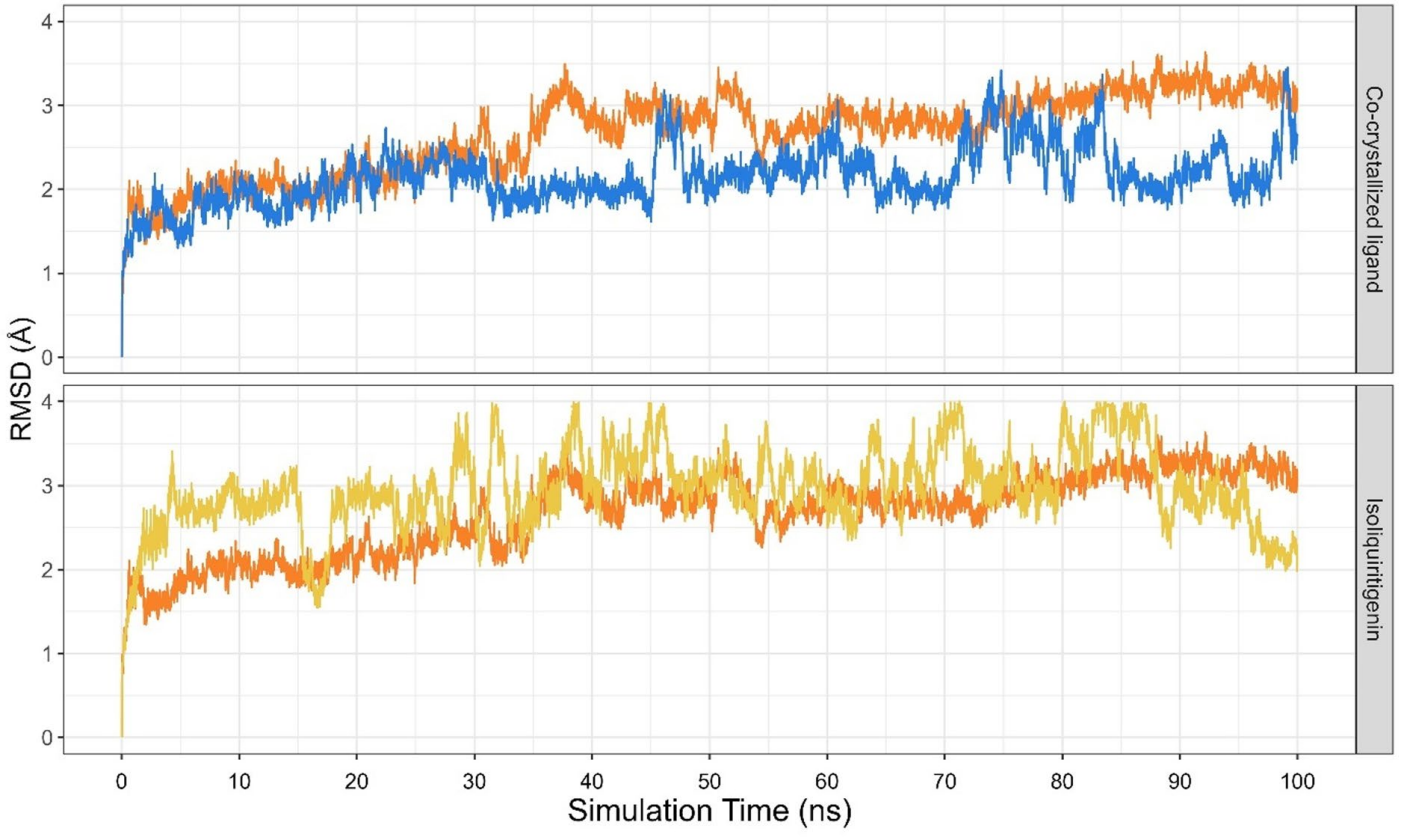
RMSD Plot of EGFR in Apo form (orange), EGFR- isoliquiritigenin complex (yellow), and EGFR-co-crystallized ligand complex (blue).

Following molecular docking, molecular dynamics simulations were conducted to assess the conformational stability of EGFR upon binding with isoliquiritigenin. The EGFR-isoliquiritigenin complex displayed a median root mean square deviation (RMSD) of 2.932 ± 0.380 Å, which was slightly higher than that of the Apo form (2.786 ± 0.491 Å) and the EGFR-co-crystallized ligand (2.108 ± 0.278 Å) (Fig. 4). Although this value is slightly higher than that of the Apo and form (2.786 ± 0.491 Å) and the EGFR-co-crystallized ligand (2.108 ± 0.278 Å), this value falls within the generally accepted 3.0 Å range for stable protein-ligand complexes, indicating that the binding remained structurally stable throughout the trajectory^28^.

**Fig. 5:**
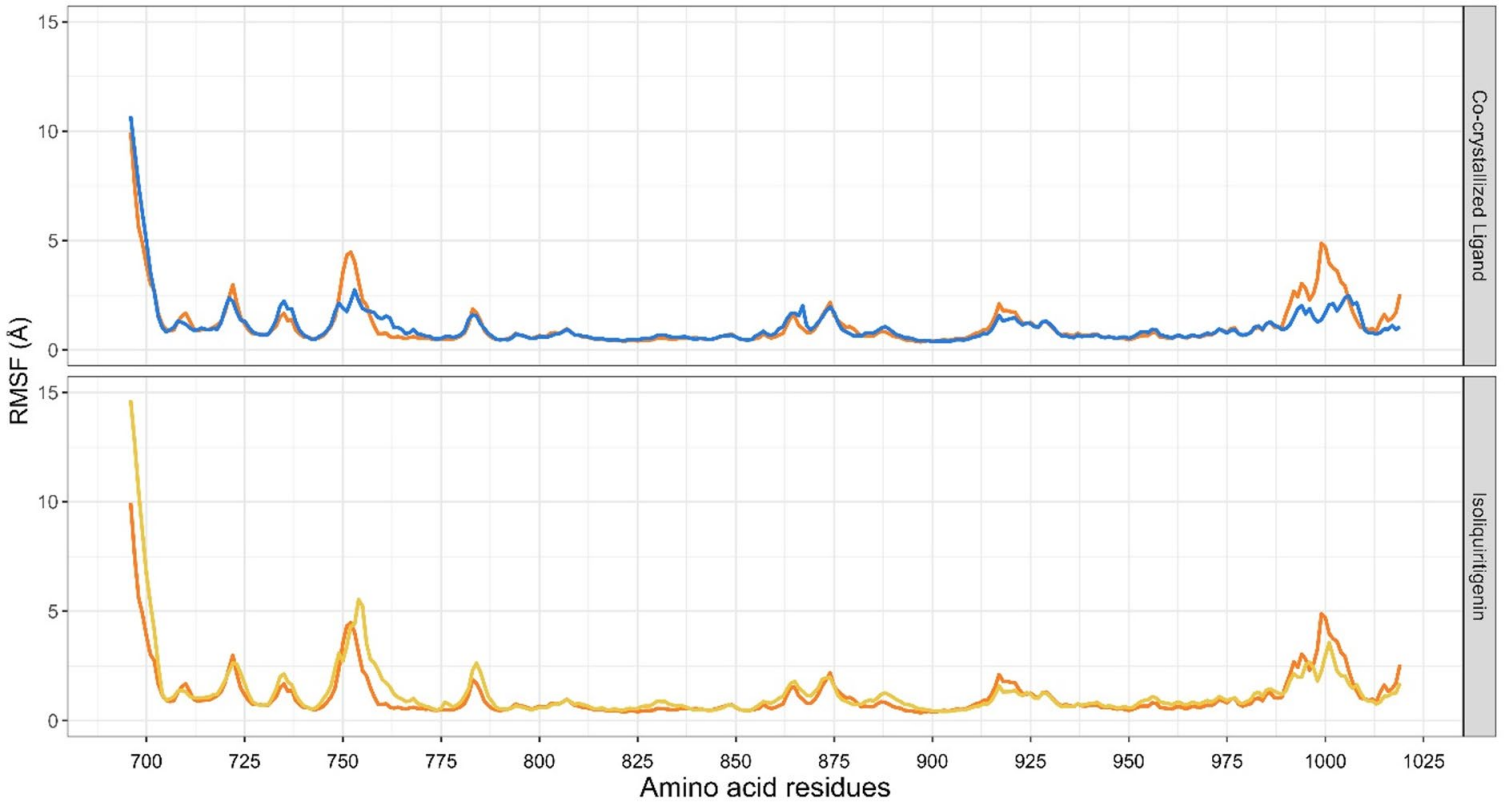
RMSF Plot of EGFR in Apo form (orange), EGFR- isoliquiritigenin complex (yellow), and EGFR-co-crystallized ligand complex (blue).

The root means square fluctuation (RMSF) plots show the residue flexibility of EGFR in complex with co-crystallized ligand and isoliquiritigenin (Fig. 5). For both complexes and the apo form, experience high fluctuation on the N-terminal region around residue 690–705, which is typical for terminal and loop regions that are naturally more flexible^29^. Importantly, the RMSF profile of EGFR in complex with isoliquiritigenin closely overlaps with that of co-crystallized ligand, suggesting that isoliquiritigenin binding did not increase backbone mobility or destabilize the receptor. This supports the stability of the EGFR- isoliquiritigenin complex.

**Fig. 6:**
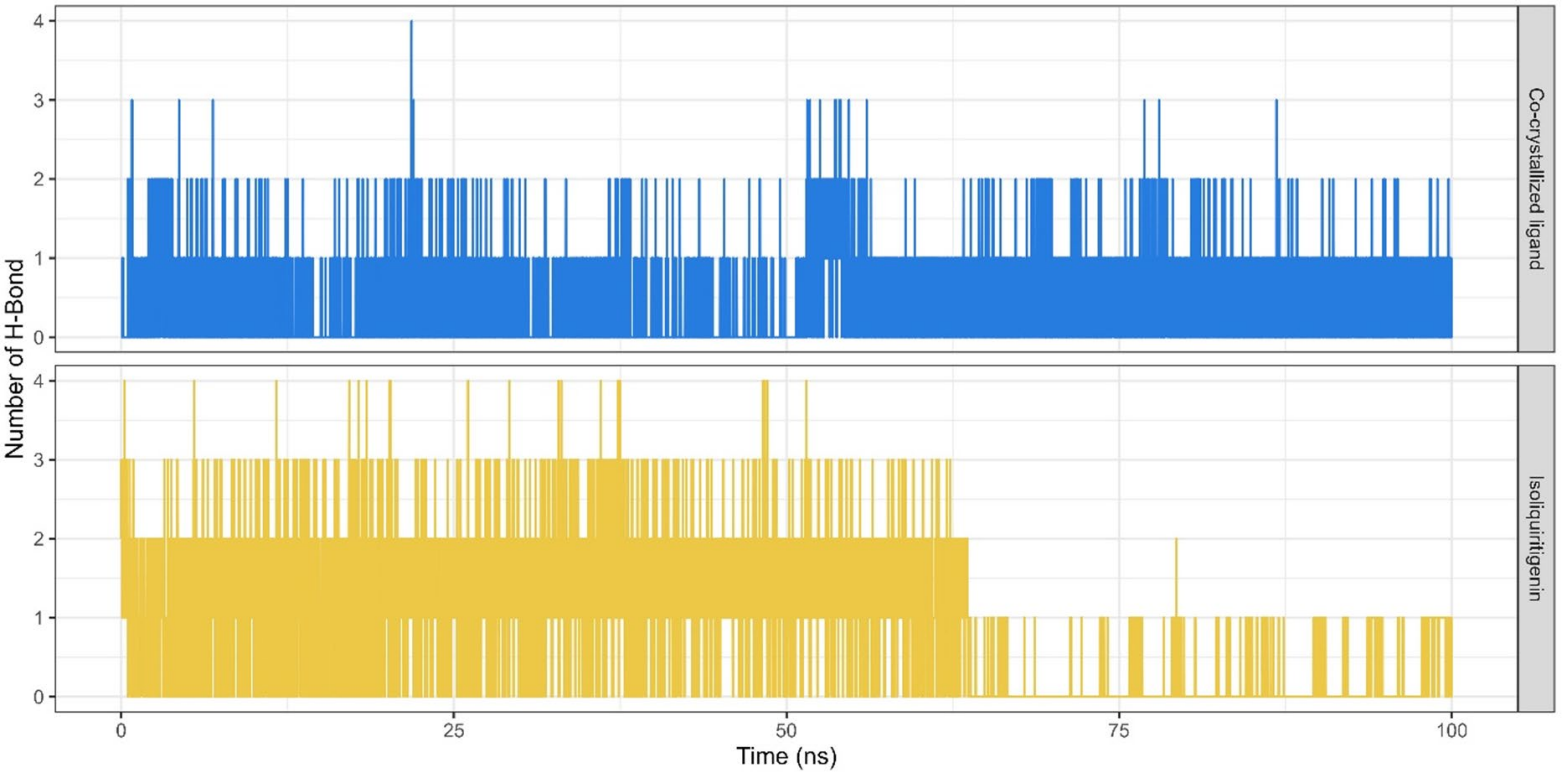
Hydrogen Bond Profile of EGFR- isoliquiritigenin complex (yellow), and EGFR-co-crystallized ligand complex (blue).

Hydrogen bonds are widely recognized as one of the most important non-covalent forces that contribute to the stability of a protein-ligand interactions^30^. In the case of the EGFR-isoliquiritigenin complex, hydrogen bond analysis revealed the consistent formation of 2–3 hydrogen bonds throughout the simulation (Fig. 6). Isoliquiritigenin form hydrogen bonds with several amino acids within the EGFR active site, including Cys775 with occupancy rates of 50.55%, Met793 (9.02%), Asp855 (6.44%), and Lys745 (3.04%). The presence of multiple hydrogen-bond donors and acceptors across these residues highlights the ability of isoliquiritigenin to establish a robust interaction network, thereby enhancing its binding affinity and potentially contributing to its inhibitory activity.

**Fig. 7:**
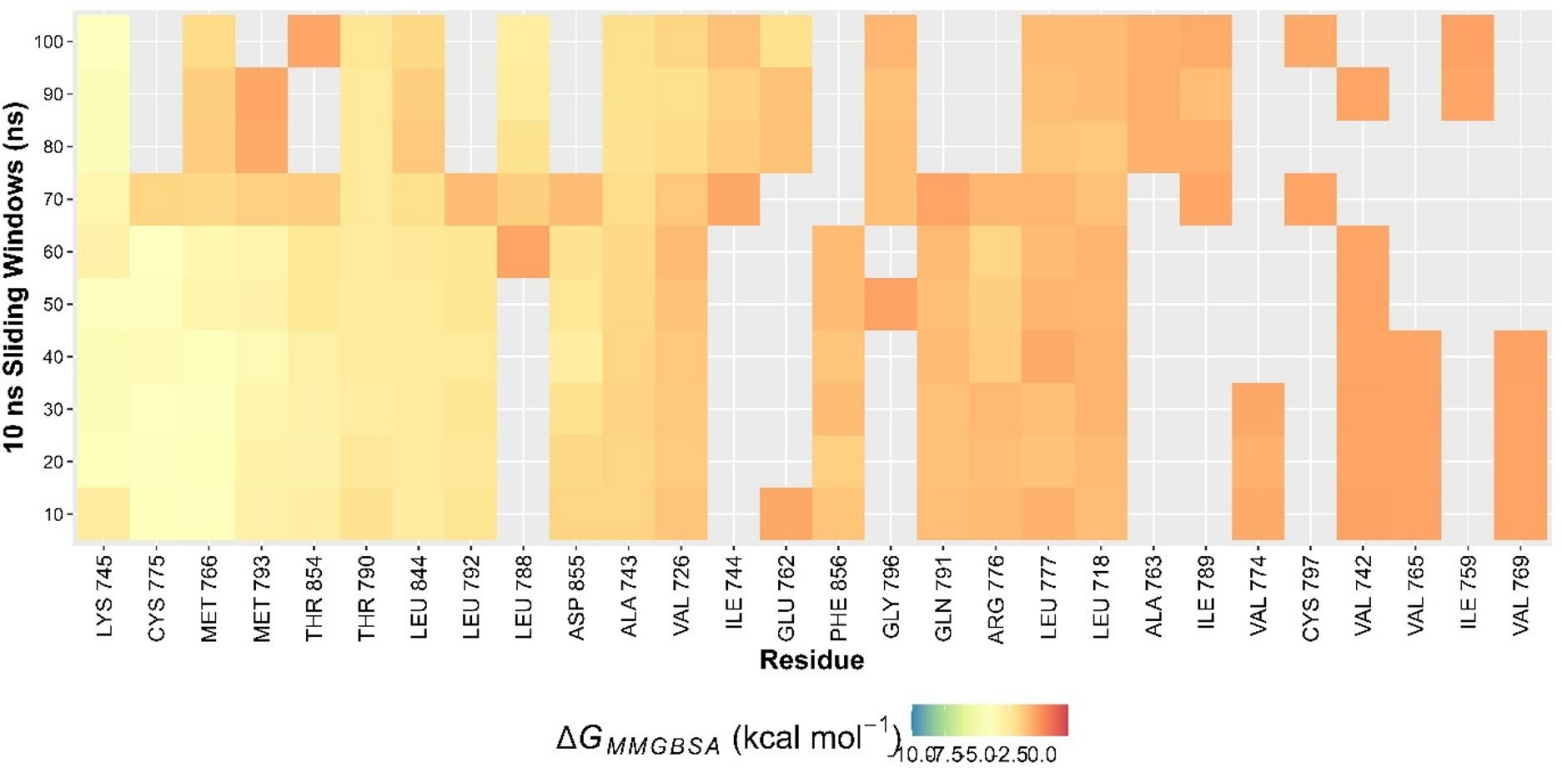
Per-residue Energi Decomposition of EGFR-isoliquiritigenin complex.

MMGBSA free energy calculation revealed that the binding energy of EGFR-isoliquiritigenin was − 30.002 ± 3.879 kcal/mol. Although this is weaker than that of the EGFR-co-crystallized ligand complex (−44.183 ± 5.431 kcal/mol), the negative value confirms that isoliquiritigenin forms a stable and favorable interaction with EGFR. Binding was mainly stabilized by van der Waals (−31.979 ± 3.331 kcal/mol) and electrostatic (−17.899 ± 6.214 kcal/mol) contributions, which outweighed the destabilizing effect of solvation (23.438 ± 3.487 kcal/mol). This result suggested that isoliquiritigenin achieves stable binding through complementary hydrophobic and electrostatic interactions. Furthermore, per-residue energy decomposition (Fig. 7) revealed that amino acid residues, including Lys745 (−4.170 ± 0.271 kcal/mol), Cys775 (−3.797 ± 0.414 kcal/mol), Met766 (−3.418 ± 1.211 kcal/mol), Met793 (−3.1645 ± 0.473 kcal/mol), Thr854 (−1.531 ± 0.952 kcal/mol), and Thr790 (−2.574 ± 0.318 kcal/mol), contributed to the binding stability of the EGFR-isoliquiritigenin complex. This finding is supported by the literature, which reports that isoliquiritigenin can interact with and inhibit EGFR activation^31,32^.

### Toxicity and pharmacokinetics prediction

The combined in vitro and in silico findings, reinforced by previous studies, support the potential of isoquiritigenin as a promising natural cytotoxic compound. To further explore its drug-likeness, the toxicity and pharmacokinetic profile of isoliquiritigenin were predicted using publicly available web servers. In terms of toxicity, isoquiritigenin was predicted to exhibit a low risk of hERG blockers, indicating a minimal likelihood of interfering with cardiac ion channels that could lead to heart arrhythmias^33^. In addition, isoquiritigenin has a low risk of being mutagenic, suggesting that it is unlikely to cause genetic mutations. However, isoquiritigenin presents a medium risk of drug-induced liver injury and a high risk of human hepatotoxicity, indicating its potential for liver damage when administered at certain dosages or over extended periods. The predicted hepatotoxicity risk of isoliquiritigenin is supported by the previous study, reporting that isoliquiritigenin exert hepatotoxicity effect by inducing endoplasmic reticulum (ER) stress mediated injury in zebrafish embryos^34^. The pharmacokinetic predictions of compound 3, including its absorption, distribution, metabolism, and excretion properties, are presented in Table 1.

**Table 1:** Pharmacokinetics prediction of isoliquiritigenin.

Pharmacokinetic property	Parameters	Predicted values
Absorption	Caco-2 permeability	–4.75 log cm/s
	Human Oral Bioavailability 50%	Bioavailable
	P-Glycoprotein Inhibitor	No
	P-Glycoprotein Substrate	No
	Human Intestinal Absorption	Absorbed
Distribution	Plasma Protein Binding	55.7
	Steady State Volume of distribution	0.83 log VDss
	Blood Brain Barrier Penetration	Non penetrable
	Fraction unbound	0.76
Metabolism	CYP1A2 inhibitor	Inhibitor
	CYP1A2 substrate	Non substrate
	CYP2C19 inhibitor	Inhibitor
	CYP2C19 substrate	Non substrate
	CYP2C9 inhibitor	Inhibitor
	CYP2C9 substrate	Non substrate
	CYP2D6 inhibitor	Inhibitor
	CYP2D6 substrate	Non substrate
	CYP3A4 inhibitor	Inhibitor
	CYP3A4 substrate	Non substrate
Excretion	Clearance	4.73 log (ml/min/kg)
	Half-life	< 3hs

Pharmacokinetic predictions provided insights into the drug-likeness and therapeutic potential of isoliquiritigenin. In terms of absorption, the compound exhibited moderate Caco-2 cell permeability (–4.75 log cm/s), but was predicted to be orally bioavailable, with good intestinal absorption. Importantly, isoliquiritigenin was not identified as either a substrate or an inhibitor of P-glycoprotein, suggesting a low likelihood of efflux-related absorption^35^. Isoliquiritigenin demonstrated moderate plasma protein binding (55.7%, with a relatively high unbound fraction (0.76), indicating that a substantial portion of the compound may remain freely available in the systemic circulation. The predicted steady-state volume of distribution (0.83 log VDss) further suggests moderate tissue distribution, whereas its inability to cross the blood–brain barrier indicates limited central nervous system penetration^36^, which may be advantageous for reducing off-target neurotoxicity.

The metabolism profile showed that isoliquiritigenin is not a substrate of major cytochrome P450 isoforms (CYP1A2, CYP2C19, CYP2C9, CYP2D6, and CYP3A4), but was predicted to act as an inhibitor of these enzymes, which is in line with previous literature^37^. For excretion, the predicted clearance rate was 4.73 log (ml/min/kg), consistent with efficient elimination, and the half-life was estimated to be less than 3 h, suggesting relatively rapid clearance from the body. Although this may limit prolonged systemic exposure, it also reduces the risk of accumulation and the associated toxicity. The broad CYP inhibition of isoliquiritigenin can increase the risk of drug-drug interaction and contribute to hepatotoxicity by disrupting normal metabolic clearance^38^. Combined with the hepatotoxicity risk, these findings highlight the need for careful safety evaluation during preclinical development. Strategies such as structural optimization or formulation approaches that limit systemic exposure like encapsulation can help mitigate this metabolic liability^39^. 

### Limitation and perspectives

Although this study provides useful insight into the anticancer potential of flavonoids isolated from *E. crista-galli*, several limitations should be acknowledged. The cytotoxic evaluation was limited to a single cancer cell line. While the in silico study provided supportive explanation of the anticancer mechanism, experimental studies to assess apoptosis pathways, ROS generation, or cell-cycle effects are still needed to help clarify the underlying mechanism. Furthermore, structural optimization of isoliquiritigenin and exploration of its synergetic effects with standard chemotherapeutics may also provide promising oportunities for the development of more effective EGFR inhibitors.

## Conclusion

This study highlights *Erythrina crista-galli* as an important reservoir of bioactive flavonoids, with promising anticancer potential. The successful isolation of naringenin, daidzein, and isoliquiritigenin underscores the phytochemical richness of this species. The notable in vitro activity of isoliquiritigenin against MCF-7 breast cancer cells, supported by computational studies, demonstrates its potential as a natural anticancer candidate. Further investigations, including in vivo validation, comprehensive toxicity assessment, and structural optimization, are necessary to fully uncover its therapeutic potential. Collectively, these findings suggest that *E. crista-galli* is a valuable natural source for the discovery and development of novel anticancer agents.

## Supplementary Information

Below is the link to the electronic supplementary material.

Supplementary Material 1

## Data Availability

All data generated or analysed during this study are included in this published article.
