# Supplementary material for: In vitro and in silico evaluation of flavonoids from Erythrina crista-galli with cytotoxic potential against MCF-7 breast cancer cell
Source: Sci Rep. 2025 Dec 18;16:2753. doi: 10.1038/s41598-025-32400-4 (PMC12824246; doi:10.1038/s41598-025-32400-4)
Supplement: Supplementary file 1 — Supplementary Material 1 [file 41598_2025_32400_MOESM1_ESM.docx]

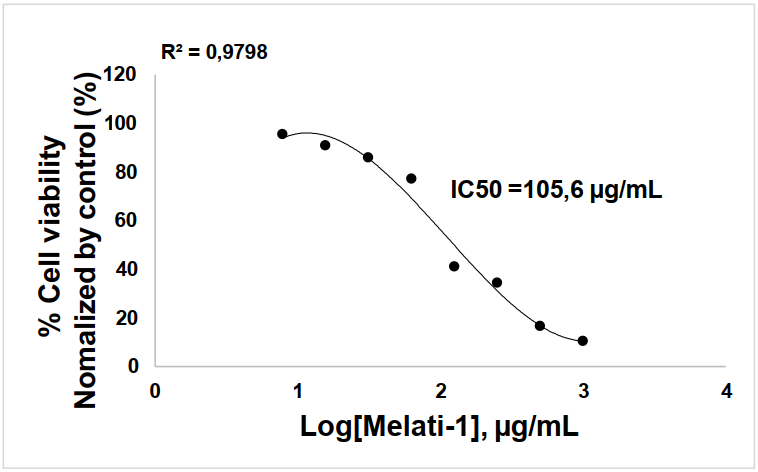


Figure S1. Sigmoid curve showing the dose-dependent cytotoxicity of compound 1 against MCF-7 cancer cell line


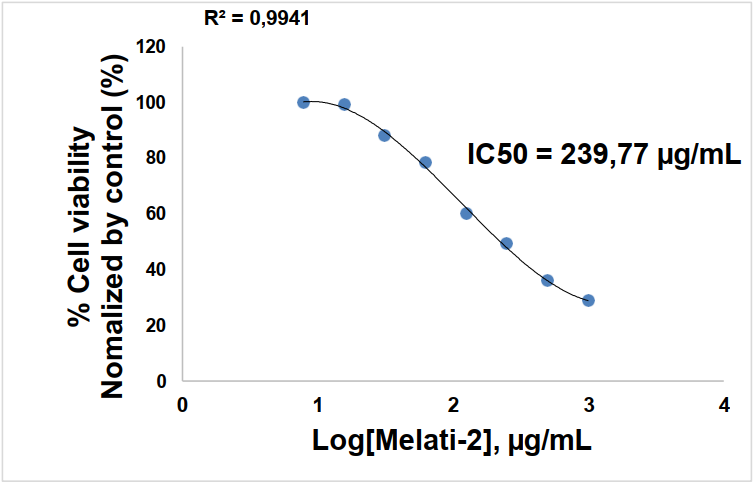


Figure S2. Figure S1. Sigmoid curve showing the dose-dependent cytotoxicity of compound 2 against MCF-7 cancer cell line


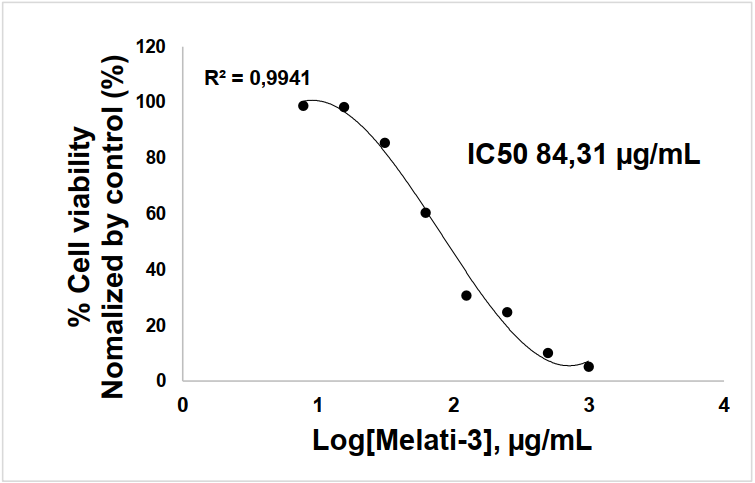


Figure S3. Figure S1. Sigmoid curve showing the dose-dependent cytotoxicity of compound 3 against MCF-7 cancer cell line


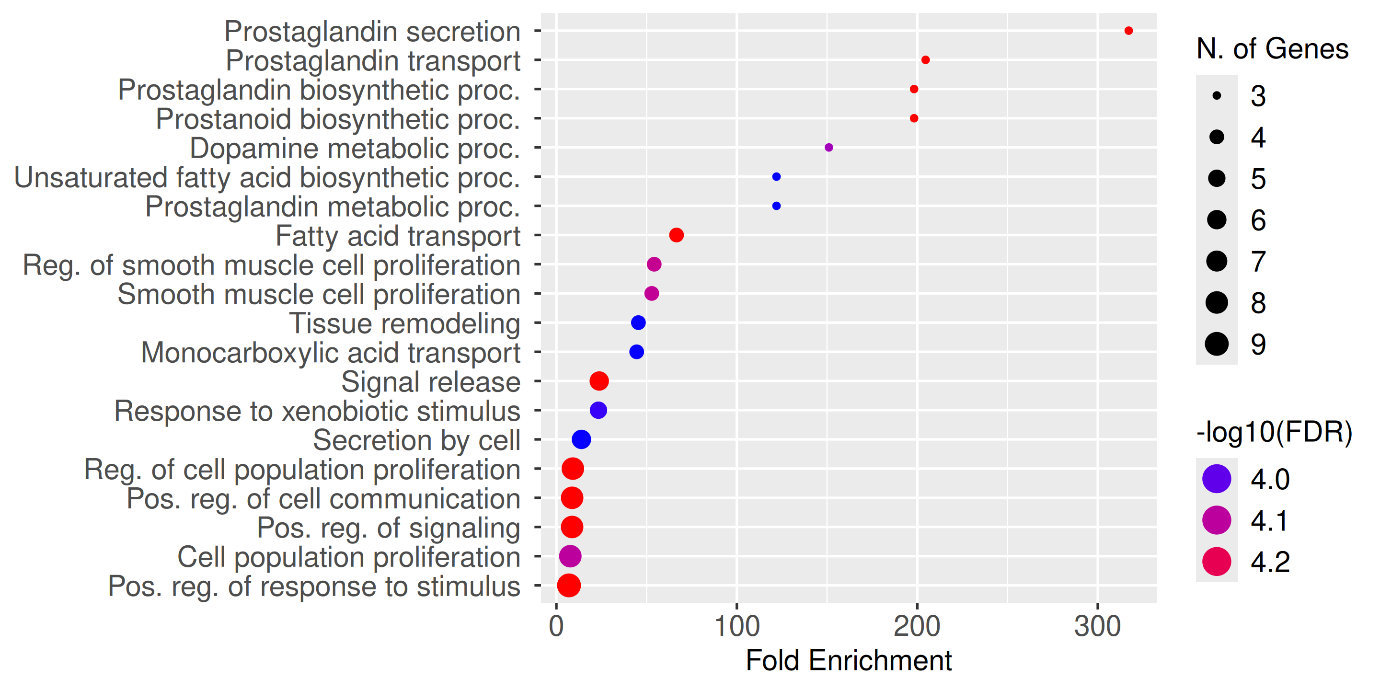


Figure S4. Gene ontology with reference to biological processes


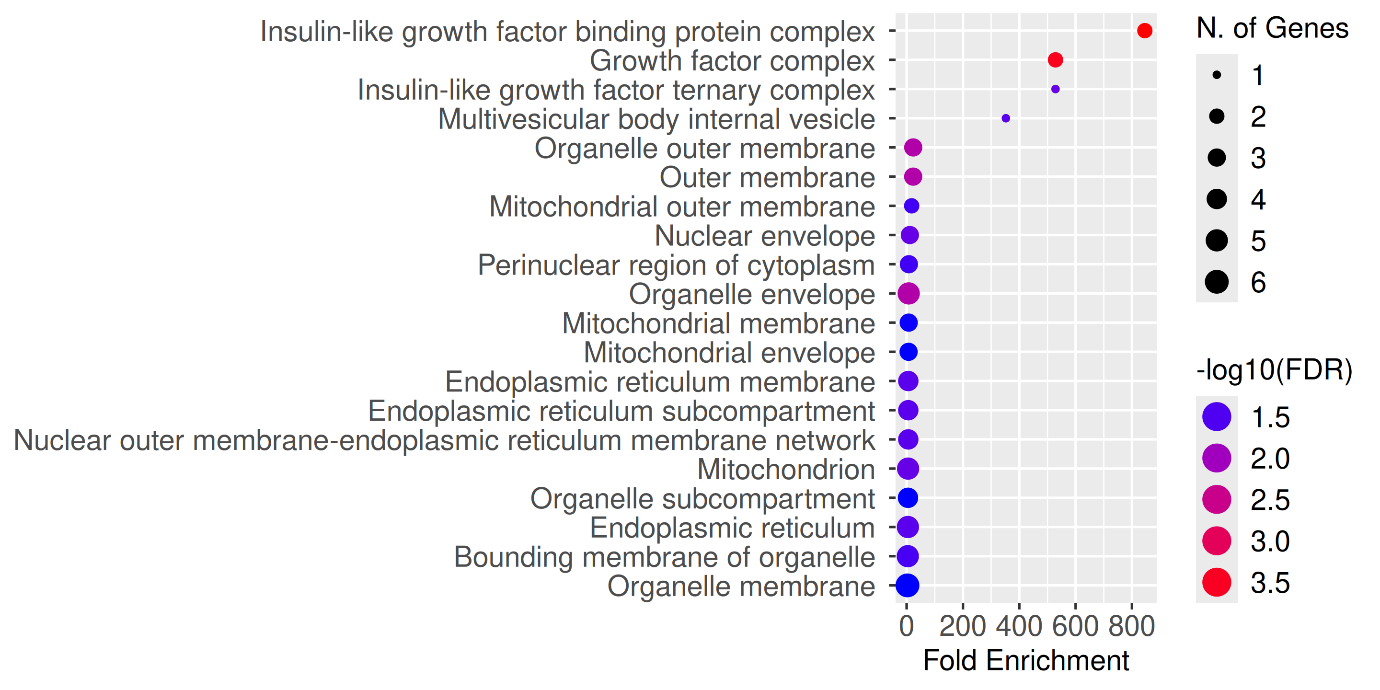


Figure S5. Gene ontology with reference to cellular component


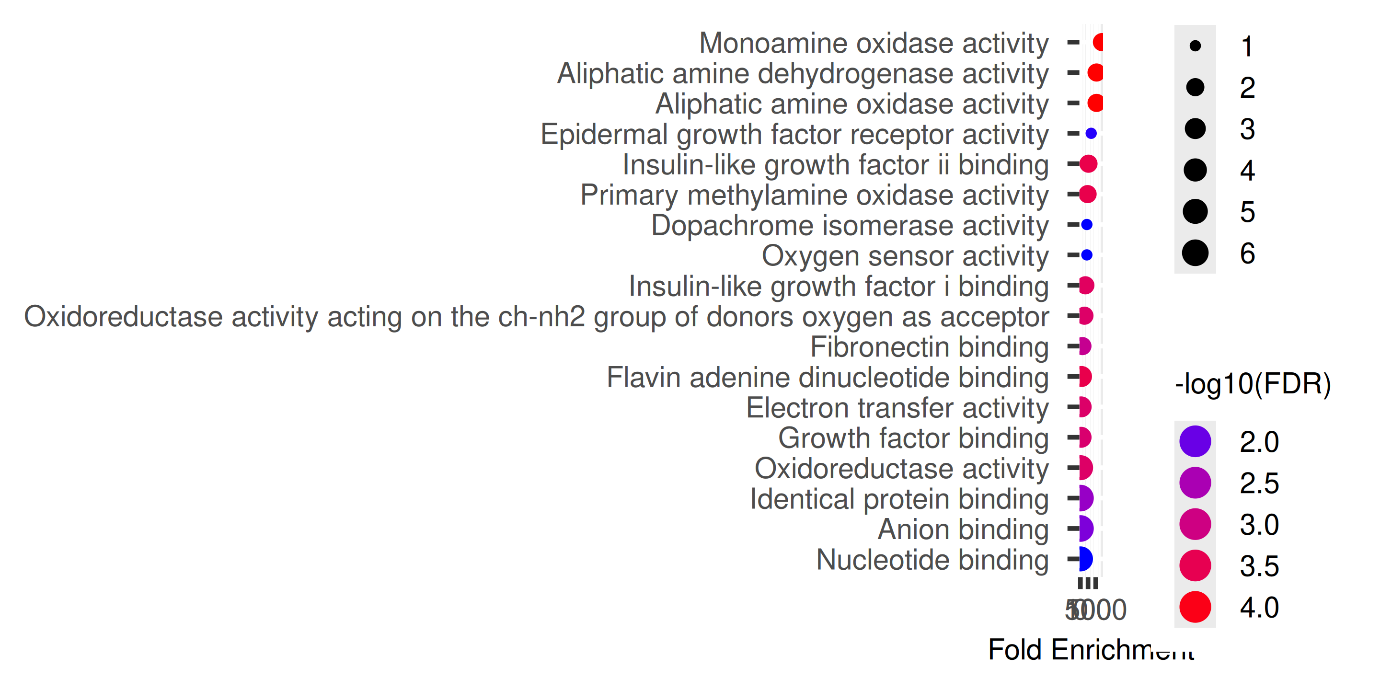


Figure S6. Gene ontology with reference to molecular function


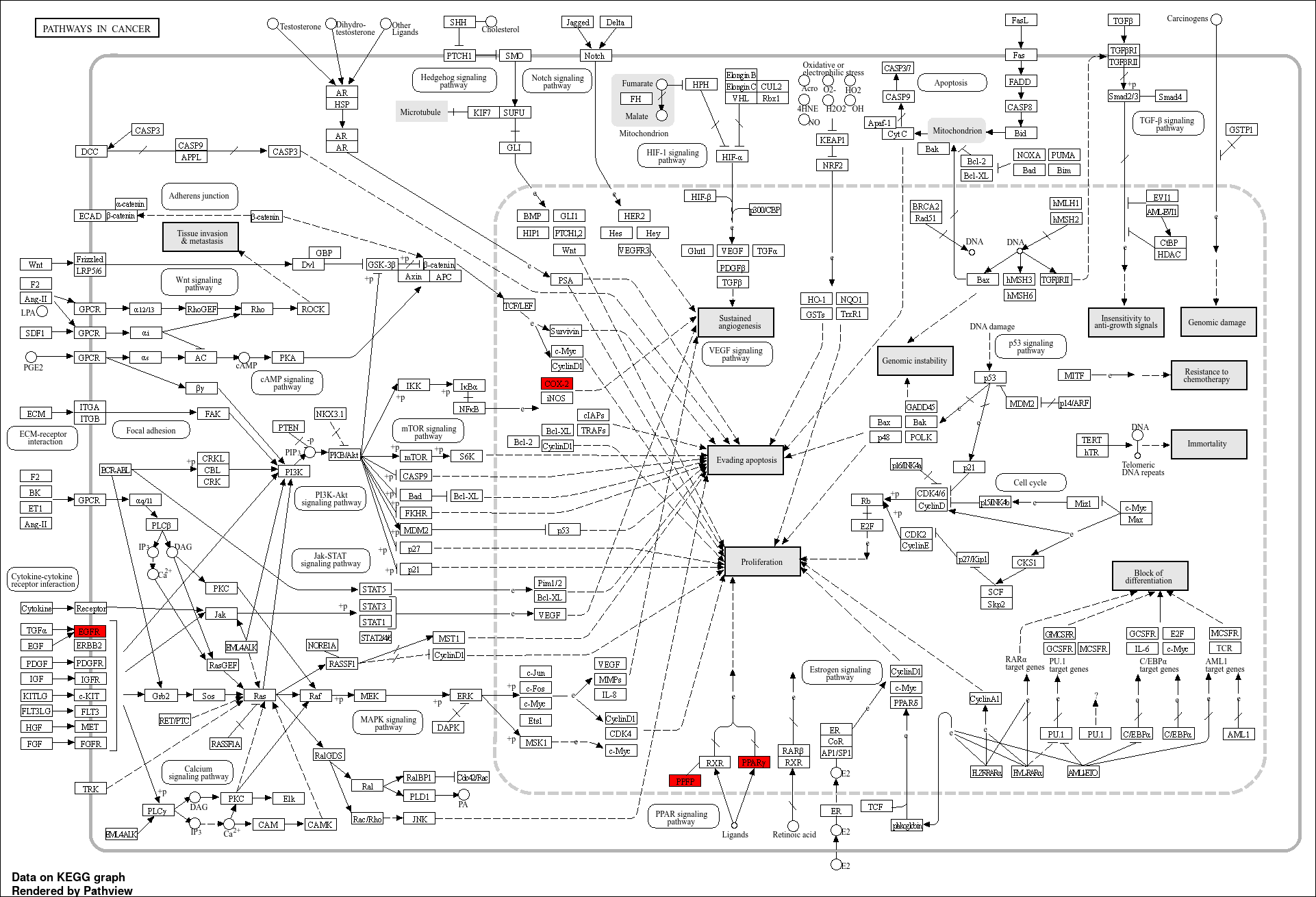


Figure S7. Kyoto Encyclopedia of Genes and Genomes (KEGG) involved in the cancer related pathways of isoliquiritigenin adapted from KEGG pathway map [1]. Permission obtained from KEGG

**Reference**

1. Kanehisa, M. KEGG: Kyoto Encyclopedia of Genes and Genomes. *Nucleic Acids Res* 28, 27–30 (2000).
